# Schemes for Co-Operation

**Published:** 1920-07-31

**Authors:** 


### HOSPITALS AND POOR-LAW INFIRMARIES.

### Schemes for Co-operation.

A proposal has been made for co-operation between King's College Hospital and the Camberwell Poor-Law Infirmary by linking the two institutions for the specialised treatment of patients and teaching of students. In submitting this scheme to the Camberwell Board of Guardians the authorities of King's College Hospital point out that, while the hospital would gain teaching advantages, the Guardians would, without cost to the borough, gain the services of eminent specialists for the patients in the infirmary.

In this connection, such a scheme has been arranged between the Paddington Infirmary and St. Mary's Hospital, and a statement by the Paddington Guardians shows that the scheme was approved on the following conditions :

(*a*) That the ordinary work of the infirmary is not interfered with.

(*b*) That the conditions are under the control of the medical superintendent.

(*c*) That the scheme be subject to termination when so decided by the Guardians.

So far the scheme has worked satisfactorily, both from the hospital and the infirmary point of view, and the Ministry of Health has sanctioned it. St. Mary's Hospital has adopted the scheme of paid "Surgical" and "Medical" Units, and the consultants (to the Infirmary) in those branches belong to these units.

Dr. E. W. G. Masterman, the medical superintendent of the infirmary, was asked to give his views on the proposal, and he reports as follows :

"King's College Hospital, in bringing up the subject of our co-operation with them before the Guardians, have anticipated my own action.

"I think all the infirmary authorities are coming to realise that the old system of looking after the patients by means of a superintendent and several junior assistant officers is not satisfactory, and I hear, in Poor-Law infirmaries all round London, arrangements are now being made which in the case of several infirmaries will mean association of the infirmary with a teaching school of medicine.

"The Guardians will undoubtedly have to face one of two alternatives : the first is to make the Infirmary a complete self-contained hospital with special departments. This will run into some thousands of pounds extra expenditure annually without doubt. (At Lambeth, for example, where there is a fairly complete pathology department, there is a whole-time pathologist who receives £500 a year, and the equipment of the department must cost probably another £500 a year. Our *x*-ray installation is utterly inadequate for modern methods, and the full methods of *x*-ray work require the attendance of a specialist. These are only two examples of what it would be necessary to do if we tried to work the infirmary as an independent unit.)

"The other proposal, which has been carried out elsewhere, is that of associating the infirmary with a teaching-school, whereby in return for the use of pathological, radiographic, and other specialised departments we should offer to the medical school, in this case King's College Hospital, opportunities to their staff to use the material in our Infirmary for teaching. Personally, I believe in such a plan, if properly arranged.

"I may say that I have on many occasions had the benefit of expert advice from King's College Hospital, which has been ungrudgingly given."

The Infirmary Committee recommends the Board to adopt the principle of co-operation.

